# IL28B rs12979860 genotype as a predictor marker of progression to BKVirus Associated nephropathy, after kidney transplantation

**DOI:** 10.1038/s41598-017-06915-4

**Published:** 2017-07-27

**Authors:** Roee Dvir, Vera Paloschi, Filippo Canducci, Giacomo Dell’Antonio, Sara Racca, Rossana Caldara, Giuseppe Pantaleo, Massimo Clementi, Antonio Secchi

**Affiliations:** 1 Laboratory of Clinical Microbiology & Virology, San Raffaele Hospital IRCCS, Milan, Italy; 2 Transplant Unit, Department of Internal Medicine, San Raffaele Hospital IRCCS, Milan, Italy; 3 Department of Pathology, San Raffaele Hospital IRCCS, Milan, Italy; 4 University of Insubria, Dept. of Biotechnology and Life Sciences, Varese, Italy; 5 UniSR-Social.Lab [Research Methods], Faculty of Psychology, Vita Salute San Raffaele University, Milan, Italy; 6 Vita-Salute San Raffaele University, Milan, Italy

## Abstract

BK virus (BKV) associated nephropathy (BKVAN) is still an important cause of allograft dysfunction after kidney transplantation (KT). Recent data have shown that the new interferon (IFN)-λ family has been ascribed antiviral properties similar to IFNα, and that the response to IFNλ in kidney is restricted to epithelial cells, suggesting that the IFNλ system evolves as specific protection of the epithelia. We aimed to test the hypothesis of correlation between a single nucleotide polymorphism (C/T dimorphism rs12979860) in the genomic region of IL28B and BKVAN, in patients after KT. Fifty kidney-transplanted patients were included as follow: *Group 1 (BKV*+*/BKVAN*+*)*: 11 patients with active BKV− replication and biopsy-proven BKVAN; *Group 2 (BKV*+*/BKVAN*−*)*: 22 patients with active BKV− replication but without evidence of BKVAN; *Group 3 (BKV*−*/BKVAN*−*):* 17 patients without evidence of BKV− replication (control group). Here we show that the C/C genotype was statistically higher in group 2 than in group 1 and BKVAN was detected significantly more frequently in patients with C/T and T/T genotypes than in patients with C/C genotype. We therefore propose IL28B polymorphism (rs12979860), as a predictor-marker to differentiate between patients with self-limited, even if persistent, BKV− reactivation and patients with a high risk of progression towards BKVAN, and to modulate the clinical management of these patients accordingly.

## Introduction

Two decades after first being reported, BKVAN remains an important cause of allograft dysfunction and graft loss after KT^1–5^, with reported rates of graft loss range from 10% to 80%. Although there has been a significant increase in clinical awareness resulting in earlier BKV diagnosis, only limited improvements regarding graft loss due to BKVAN have been accomplished. Many studies have described BKVAN risk factors^6–9^, effective BKV screening methods^10–15^, and early predictive bio-markers for the evolvement of BKV infection into BKVAN^16–19^. Furthermore, our understanding of the unique characteristics of BKV infection in renal tubular epithelial cells and urothelial cells has improved in recent years^20, 21^, with clear differences having been shown between human urothelial cells (HUCs) and renal proximal tubular epithelial cells (RPTECs), both infected by BKV. In HUCs, the BKV replication cycle is slower with less viral progeny being released. However, the most important observation has been the cytopathogenic effect, resulting in massive cell detachment of HUCs, without cell lysis. It is presumed that detachment, rather than cell lysis, might explain the absence of significant inflammation, despite the high level of BKV replication in the urothelium. Moreover, new information regarding BKV− specific immunity kinetics^22^, as well as activation of the antiviral state by the polyomavirus T antigen, is now available, providing testable hypotheses on how BKV replication may cause severe disease in humans, and the response of innate immune activation after viral infection^23, 24^. In particular, Nicholas *et al*.^25^ demonstrated that the BKV T-antigens (BKV trunk T antigen and most probably the BKV small T antigen) are able to drive an antiviral state by activating the cellular IFN-response, and therefore ISGs up-regulation, in a STAT1-dependent mechanism. The ISGs can interfere at almost any step of the viral life-cycle, and are associated with antiviral activity.

The IFNλ family consists of IL29 (IFNλ1), IL28A (IFNλ2) and IL28B (IFNλ3), and binds a cell-surface receptor composed of two chains - IL10R2 and IL28R1^26, 27^. The potential interaction between these IFNs has been revealed in a genome-wide association study, in which a single nucleotide polymorphism (C/T dimorphism rs12979860) in the genomic region of IL28B was strongly associated with sustained viral response (SVR) in hepatitis C virus (HCV) patients treated with pegylated-IFNα^28^. Patients homozygous for the protective allele (C/C) had a significantly higher SVR than patients carrying the risk allele T, both in the homozygous and in the heterozygous forms^28, 29^. To-date, IL28B polymorphism is the strongest pre-treatment predictive bio-marker for the success of peg-IFNα therapy in chronic HCV patients. The protective IFNλ allele, in the setting of HCV infection, is thought to mediate viral clearance through immune activation or direct antiviral effects via the stimulation of specific ISGs. In fact, type I (IFNα/β) and type III IFNs are often considered the antiviral classes, and each of these induces a unique and partially overlapping set of ISGs. Several studies have explored the feedback mechanisms between IFNλ and IFNα, which might be affected by the SNP rs12979860, and, therefore, might influence virus replication and IFNα therapy efficacy in diseases other than viral hepatitis^30^. New data on IFNλ and the demonstration of the high-level of IFNλ receptor expression in the urinary epithelium, BKV latency and active replication site, have recently been published^31, 32^. In particular, Sommereyns *et al*. demonstrate that the response to IFNλ in kidney is restricted to epithelial cells, suggesting that the IFNλ system evolves as specific protection of the epithelia. We raised the hypothesis of a possible correlation between IL28B polymorphism and BKVAN in patients following KT, and the possibility, therefore, to use IL28B polymorphism to differentiate between patients with self-limited BKV− reactivation and those prone to developing BKVAN.

## Results

Overall, 50 Caucasian adult after KT, kidney-pancreas transplantation, or double kidney transplantation, who were transplanted or followed at our institute between 1997 and 2012 were included in our single-center retrospective case-control genetic association study. Thirty-three patients with active BKV− replication, determined by BKV− DNA viruria and/or viremia, and 17 patients with no BKV− reactivation were included and assigned to three different groups as follows: *Group 1 (BKV*+*/BKVAN*+): 11 patients with active BKV− replication and biopsy-proven BKVAN; *Group 2 (BKV*+*/BKVAN*−*)*: 22 patients with active BKV− replication and allograft biopsy negative for BKVAN; *Group 3 (BKV*−*/BKVAN*−*):* 17 patients without BKV replication/infection (control group). In the latter group, biopsies were performed for clinical reasons (worsening allograft function). Patient history, clinical and histological and laboratory data were collected and analyzed. Demographic and baseline determinants of the different groups are summarized in Tables 1 and 2:

**Table 1:** Demographic and clinical basal characteristics of the 3 groups.

	Group 1*BKV*+/BKVAN+ (11 pt.)	Group 2BKV+/BKVAN− (22 pt.)	Group 3 BKV−/BKVAN− (17 pt.)	P Value
Recipient				
*Gender (%)*				
Men	8 (73%)	15 (68%)	10 (59%)	NS
Women	3	7	7	NS
*Mean Age (years)*	56 (28–69)	49 (34–66)	45 (31–68)	NS
*Cause of ESRD*				
DM	54.5%	45.5%	23.5%	NS
GNF	27.3%	22.7%	47%	NS
Other	18.2%	31.8%	29.5%	NS
*Transplant*				
Kidney *(live donor)*	5 (0)	10 (2)	15 (3)	NS
DKT	3	4	0	NS
KPT	3	8	2	NS
*Cold ischemia time (hours)*	12.95	13.5	10.62	NS
Donor				
*Mean Age (years)*	59 (17–74)	45 (16–75)	51 (23–76)	NS
*Weight (Kg)*	71.2	67.4	70.46	NS

Abbreviations: ESRD – End Stage Renal Disease, DM – Diabetes Mellitus, GNF - Glomerulonephritis, DKT - Double Kidney Transplantation, KPT - Kidney-Pancreas Transplantation.

**Table 2:** Immunosuppression and clinical outcome of the 3 groups.

	Group 1BKV+/BKVAN+ (11 pt.)	Group 2 BKV+/No BKVAN (22 pt.)	Group 3 No BKV (17 pt.)	P Value
HLA Mismatch number	2.2	3.4	3.5	0.027
ATG induction	63.6%	81.8%	52.9%	NS
IS maintenance:				
MMF	72.7%	81.8%	70.5%	NS
Prednisone	63.6%	54.5%		NS
Tacrolimus	90.9%	68.2%	82.3%	NS
CSA	9.1%	22.7%	17.6%	NS
Sirolimus/Everolimus	27.3%	13.6%	41.1%	NS
Acute rejection (%)	45%	36.3%	47%	NS
Cell Mediated	60%	87%	75%	
Antibody Mediated	40%	13%	25%	
Creatinine clearance (ml/min/1.73mq)	Mean 28.7 (13–56)	Mean 55.8 (17–138)	Mean 35.4 (8–94)	0.006
CMV infection	27.3%	31.8%	29.4%	NS
Months from transplantation to BKV− first detection*	9	26		0.011
Viruria (copies/ml, Log)	7.0 (0.31)	6.0 (1.62)		NS
Viremia (copies/ml, Log)	5.0 (0.93)	3.39 (1.35)		0.003

Abbreviations: BKV – BK virus, BKVAN – BK Virus Associated Nephropathy, IS – immunosuppression, MMF - mycophenolate mofetil, CSA - cyclosporin A, CMV- cytomegalovirus.

*Months from transplantation to BKV− first detection – calculation was made from 2009 (introducing BKV real-time PCR quantitative assay) for patients who were transplanted before that date.

*Group 1* - included 11 cadaver kidney recipients with biopsy-proven BKVAN. Mean time between transplantation and BKV− reactivation was 9 months (range, 1–32 months), which was significantly lower than in those patients without evolution to BKVAN (*p* = *0.011*). Overall, 72% of the patients in this group were documented with BKV− reactivation during the first post-transplantation year. All patients have sustained BKV viruria, with a mean BKV− DNA viral load of 7.00 ± 0.31E copies/mL, whereas all but 2 patients have sustained BKV viremia, with a mean viral load of 5.00 ± 0.93E copies/mL. The mean estimated glomerular filtration rate (eGFR) at the time of BKV− reactivation and biopsy performance, according to the Modification of Diet in Renal Disease (MDRD) Study equation, was 28.7 ± 13.4 ml/min/1.73mq. Three (27%) patients lost their allograft due to BKVAN and returned to dialysis. Overall, 5 (45.5%) patients experienced an episode of acute rejection (1 before the diagnosis of BKVAN and 4 at the same time as BKVAN diagnosis), as follow – 3 patients have had cell-mediated rejection (CMR) and 2 patients have had Ab-mediated rejection.

*Group 2* – included 20 cadaver-kidney recipients and 2 live-kidney recipients with active BKV− replication, but with no diagnosis of BKVAN at allograft biopsy. Donor mean-age (45 yr) was lower, however not significantly, than in group 1 (59 yr), although both had the same age range. The absence of BKVAN was histologically proven by a negative biopsy in 16 patients, while for 6 patients the absence of nephropathy was based on confirmed stability of graft function. Mean BKV viruria was 6.00 ± 1.62E copies/mL, lower than in group 1 but with no statistically significant difference, whereas mean BKV viremia was 3.39 ± 1.35E, significantly lower than in group 1 (*p* = *0.003*). Seven (32%) patients had sustained BKV viruria and 7 (32%) patients had sustained BKV viremia. Mean creatinine clearance at the time of BKV− reactivation was 55.8 ± 27.6 ml/min/1.73mq, significantly higher than in group 1 (*p* = *0.006*). Mean time to BKV− first detection was 26 months, significantly longer than in group 1 (*p* = *0.011*). Overall, 3 (13.6%) patients lost their graft: one patient because of acute rejection after immunosuppression reduction due to severe side-effects; one patient due to Page syndrome complicating allograft biopsy, and one patient due to chronic rejection. Kidney allograft rejection was diagnosed in 8 patients (36%; 7 patients with CMR and only one patient with AbMR) as follow: in 5 cases before BKV− reactivation, whereas in only 1 case after BKV− reactivation. In 2 other cases, it was diagnosed at biopsy, which was performed owing to BKV− reactivation.

*Group 3 -* the control group included 17 patients without any sign of active BKV− replication neither in urine nor in plasma. All patients underwent allograft biopsy for clinical reasons, and acute rejection was diagnosed in 8 patients (47%; 6 patients with CMR and 2 with AbMR).

IL28B genotypes frequencies are shown in Table 3. Statistically significant differences were observed in group 1 and 2: the C/C genotype was statistically more frequent in group 2 than in group 1, and BKVAN was detected significantly more frequently in patients with minor-allele genotypes (C/T and T/T) than in patients with major-allele genotype (C/C) (*p* = *0.026*). The distribution of IL28B polymorphism in the control group (group 3) was similar to the general population^33^. Demographic and baseline determinants of the different groups are in Table 4.

**Table 3:** IL28B SNP rs12979860 genotype distribution in the study groups.

IL28B (SNP rs12979860)	Group 1 BKV+/BKVAN+ (11 pt.)	Group 2 BKV+/BKVAN− (22 pt.)	Group 3 BKV−/BKVAN− (17 pt.)	P Value (group 1 vs group 2)
C/C	2 (18.2%)	14 (63.6%)	7 (41.2%)	0.026
C/T-T/T	9 (81.8%)	8 (36.4%)	10 (58.8%)	
C/T	7 (63.6%)	7 (31.4%)	9 (53%)	
T/T	2 (18.2%)	1 (5%)	1 (5.8%)	

Abbreviations: BKV – BK virus, BKVAN – BK Virus Associated Nephropathy.

**Table 4:** Demographic and baseline determinants of the different groups

Groups	Sex	Age (at Tx)	Tx (Live Donor)	Creatinine clearance MDRD	BKV− first detection* (months from Tx)	BKV Viruria cp/mL (duration - months)	BKV Viremia cp/mL (duration - months)	IL28B Genotype
Group 1 (BKV+/BKVAN+)								
1	F	28	KPT	41	27	4.3E + 06 (P)	2.0E + 03 (P)	C/T
2	M	60	DKT	31	4	1.0E + 07 (P)	2.6E + 04 (P)	C/T
3	M	68	DKT	24	1	1.0E + 07 (P)	4.7E + 02 (P)	C/C
4	M	42	KPT	13	16	1.0E + 07 (P)	1.0E + 06 (P)	C/C
5	M	55	Kidney	14	2	1.0E + 07	2.0E + 02	T/T
6	M	65	DKT	16	4	5.3E + 03 (P)	7.5E + 04 (48)	C/T
7	M	69	Kidney	23	2	1.0E + 07 (P)	3.5E + 05 (7)	C/T
8	M	69	Kidney	25	2	4.0E + 05 (P)	9.1E + 05 (P)	C/T
9	M	48	Kidney	43	8	NP	1.3E + 04 (24)	T/T
10	F	48	Kidney	30	32	1.0E + 07 (P)	1.2E + 05 (P)	C/T
11	F	64	KPT	56	3	6.6E + 07 (24)	1.7E + 05 (24)	C/C
Group 2 (BKV+/BKVAN−)								
1	M	56	KPT	138	24	NP	4.7E + 02 (12)	C/C
2	F	67	DKT	25	16	1.0E + 07 (P)	3.2E + 03 (P)	C/C
3	M	45	Kidney	53	6	3.7E + 05 (P)	3.1E + 05 (51)	C/C
4	F	42	Kidney	54	1	1.6E + 04 (1)	0	C/C
5	M	59	Kidney	41	23	5.0E + 04 (P)	1.7E + 04 (P)	C/C
6	M	36	KPT	35	41	NP	1.9E + 02 (1)	C/T
7	M	36	KPT	27	50	1.2E + 08 (P)	3.0E + 05 (P)	C/T
8	F	34	Kidney	33	24	9.7E + 02 (2)	2.2E + 02 (1)	C/C
9	M	45	KPT	64	21	8.0E + 06 (36)	2.1E + 05	C/C
10	M	63	DKT	68	4	3.21E + 04 (P)	1.4E + 05 (20)	C/T
11	M	56	DKT	48	3	1.0E + 07 (P)	3.2E + 05 (7)	C/C
12	F	42	Kidney	49	23	4.5E + 07 (P)	5.5E + 03 (P)	C/T
13	M	65	Kidney	27	52	1.0E + 03 (P)	3.3E + 02 (5)	C/T
14	F	41	KPT	53	54	8.7E + 03	3.5E + 01	C/C
15	F	36	KPT	58	41	8.7E + 02	9.2E + 01	C/C
16	F	40	KPT	102	54	NP	1.0E + 02	C/C
17	M	66	Kidney	48	27	2.5E + 04 (P)	1.6E + 02 (24)	T/T
18	M	54	(Kidney)	60	15	1.0E + 06 (P)	3.6E + 02 (27)	C/T
19	M	45	(Kidney)	66	3	5.0E + 05 (11)	1.0E + 03 (11)	C/T
20	M	52	Kidney	17	28	3.1E + 03 (P)	1.1E + 04 (64)	C/T
21	M	66	DKT	75	3	1.1E + 08 (P)	1.9E + 03 (P)	C/T
22	M	46	KPT	87	54	NP	1.E + 02	C/C
Group 3 (BKV−/BKVAN−)								
1	F	61	Kidney	19	—	0	0	C/C
2	M	31	(Kidney)	43	—	0	0	C/C
3	M	42	(Kidney)	29	—	0	0	C/C
4	M	68	Kidney	8	—	0	0	C/C
5	F	48	Kidney	15	—	0	0	C/T
6	M	40	Kidney	74	—	0	0	C/T
7	F	35	Kidney	13	—	0	0	C/C
8	M	59	Kidney	17	—	0	0	C/T
9	F	33	(Kidney)	31	—	0	0	C/T
10	M	68	Kidney	47	—	0	0	C/C
11	M	60	Kidney	28	—	0	0	C/T
12	F	38	KPT	94	—	0	0	C/T
13	M	41	Kidney	27	—	0	0	C/T
14	F	36	KPT	21	—	0	0	C/C
15	M	45	Kidney	78	—	0	0	T/T
16	M	44	Kidney	46	—	0	0	C/T
17	F	35	Kidney	12	—	0	0	C/T

Abbreviations: Crea – Serun Creatinine level in mg/dl, Tx – Transplantation, BKV – BK virus, BKVAN – BK Virus Associated Nephropathy, DKT - Double Kidney Transplantation, KPT - Kidney-Pancreas Transplant, Pos – Positive, Neg – Negative, Nb- No biopsy, ND – No Data, NP – Not Performed, P – Persistent, MDRD – Modification of Diet in Renal Disease.

*Months from transplantation to BKV-first detection – calculation was made from 2009 (introducing BKV real-time PCR quantitative assay) for patients who were transplanted before that date.

We further analyzed other host genetic polymorphisms near IFNL3, rs8099917 and ss469415590 (IFNL4) in most, but not all, of our patient cohort study. We found a very good correlation between rs12979860 and ss469415590 (95.5%; disequilibrium in only 2 of 45 patients), whereas of the two SNPs, rs12979860 predicts BKVAN better, and low correlation between rs12979860 and rs8099917 (80%; disequilibrium in 8 of 39 patients) – Data not shown.

Genotypes of rs12979860 SNP (C/C 0.46, C/T 0.46, T/T 0.08 frequencies) followed Hardy–Weinberg equilibrium in our study population (p = 0.868). There were no significant differences in the genotype distributions between this study group and the reference HapMap group in Caucasian population.

## Discussion

Early detection of BKV− reactivation and prediction markers for the risks of BKVAN are paramount for rapid intervention. The association of BKV viruria and viremia with the deterioration of renal allograft function has been well documented. However, no clear threshold levels for BKV viruria and/or viremia are unequivocally associated with BKVAN, still making it controversial to predict the evolution of nephropathy based on viral loads^34–38^. Persistently high BK viremia (≥4 log10/mL) is associated with a greater risk of BKVAN and graft dysfunction. Nevertheless, some studies report that lower levels of BK, either transient or persistent, do not have any significant negative impact on transplant outcome, whereas others describe cases of BKVAN even in patients with low viral load^30, 35^. In our study, no statistical difference was found regarding urine BKV viral load in patients with BKV− reactivation, whereas plasma viral load was significantly higher in patients with BKVAN (*p* = *0.003*). Furthermore, patients with persistent BKV viruria and viremia were seen in both groups. Moreover, at the time of diagnosis three of our biopsy-proven BKVAN patients had a lower BKV plasmatic viral load than the recommended cut-off point of ≥4 log10/ml. Our data support observations made by others concerning the risk of underestimating the diagnosis of BKVAN in patients with low BKV viral load, emphasizing the need for early markers associated with a predisposition towards BKVAN.

Statistical differences were not observed in our 3 groups as regards to donor age, recipient age, cold ischemia time, donor weight, acute rejection episodes, and cytomegalovirus infection. Unlike other studies, we could not find differences in immunosuppressive burden, and ATG induction was more frequently used in group 2 than in group 1, although the difference did not reach statistical significance.

According to the data above, we might suppose that factors other than immunosuppression can maximally predispose to BKV− reactivation and BKVAN evolution. Studies have already demonstrated the important role of the innate immune system in recognizing virus infection and replication^39^. Toll-like receptors (TKRs), a family of recognition receptors, protein complex like nuclear factor (NF)-ƙB and antiviral mediators (IFNs and pro-inflammatory cytokines like IL-6, IL-8 and TNFα) are all taking part in the host response and antiviral defense. Recently, studies by Ribiero *et al*.^23, 24^ have described a range of mechanisms by which the innate immune system is involved in BKV infection. They described an upregulation of the TLR3 in renal biopsies with BKVAN. Briefly, BKV− dsRNA is recognized by the TLR3 (specific dsRNA intracellular sensor, highly expressed in renal epithelial cells) which triggers the activation of the NF-ƙB and subsequently the production of IFNs, IL-6, IL-8^23^. They also described the involvement of the TNFα/TNF receptor system (induce tubular expression of IL-6 and IL-8 - relevant in BKVAN) in host’s BKV antiviral response^24^. Produced by most renal cells, in an inflammatory setting, TNFα, is relevant for both antiviral activity and immune modulation. In fact, the authors suggests that the above pathways of the innate immune system, in response to BKV infection, contributes to nephropathy rather than protect allograft kidney. The cellular IFN-response, through a signal transduction pathways, and therefore ISGs up-regulation, control viral infection, IFN expression and contributes to the host antiviral defense. Studies have demonstrated, for example, the efficacy of IFNλ, against influenza virus, respiratory syncytial virus and rotavirus. Although epithelial cells are the main target of IFNλ activity, several studies suggest that epithelial cells could also produce IFNλ. In fact, IFNλ production was observed in dendritic cells (DCs), respiratory epithelial cells, hepatocytes and variety of other cell lines.

Recently, high-level IL28B-receptor expression with good responsiveness to IFNλ has been reported in cells of the urinary epithelium, rather than in other tissues. These findings raise the question concerning the role of IFNλ in the immune response in case of BKV− infection, and the possibility that it might interfere with BKVAN progression. We found a statistically significant difference in IL28B polymorphism SNP rs12979860 distribution in our study groups (*p* = *0.026*), and correlation between IL28B polymorphism and overall BKVAN development. The protective role of the C/C genotype was endorsed by the observation that C/C patients even with high and sustained BKV viruria and viremia, did not progress to BKVAN. On the other hand, we report higher prevalence of T/T and C/T genotypes in group n° 1 compared to group n° 2 (81.8% vs. 36.4%, respectively). Finally, we found the same genotype distribution as in the general population in our control group (group 3 - patients with no evidence of BKV− reactivation)^33^. Therefore, our data suggests that the SNP rs12979860 might be considered as a predictive marker for BKVAN progression in patients with BKV− reactivation after KT. The genotype distribution was not significantly related to viral reactivation and/or levels of viruria and viremia in the two *BKV*+ groups (groups 1 and 2), suggesting that IL28B polymorphism does not affect BKV− reactivation, viral loads, and/or viral clearance. The damage at kidney level might be the consequence of the different activation of innate immune system in the two genotypes.

Limitation of our study should also be discussed. First, because of the relatively small number of patients involved in our study, particularly those affected by BKVAN, it was impossible to perform additional statistical analyses, for example, multivariate analyses of potential risk factors based on the simultaneous consideration of both genetic and non-genetic factors, to further illuminate and detail our main research findings. Nevertheless, the effects we were able to document in the present study, *despite* the relatively small sample size at our disposal, were all of a certain magnitude, with ‘effect sizes’ ranging from Cohen’s *d* = 0.65 to *d* = 1.18, corresponding to ‘medium/large’ to ‘very large’ observed effects. In this respect, our findings—though stemming from a single-centre study and, therefore, necessarily somewhat limited in terms of total sample size — could be considered as a starting point for further empirical research. New research endeavors, especially if conducted within the framework of multi-centre studies, will then have the power to assess *also* the role of potential confounding and related intervening factors, and of potential moderators and/or mediators of the reported findings, as well.

A second limitation could be the calculation regarding time from transplantation to BKV− reactivation (direct effect of the first limitation). Quantitative evaluation of BKV viral loads in plasma and urine was first introduced in our hospital in 2009. Therefore, we had to calculate time from transplantation to BKV− *first detection* (and not BKV− reactivation), starting at that point of time. We report time to BKV− first detection in our cohort to be 9 and 26 months in group 1 and 2, respectively (*p* = 0.011).

According to our data, we could suggest that patients reactivating BKV and carrying the IL28B C/T and T/T genotypes, and therefore are at higher risk for BKVAN, should undergo strict control of viremia and viruria, together with kidney biopsy, as soon as BKV− reactivation is documented. Contrarily, patients at “low risk” (patients carrying the protective C/C genotype) can benefit from a “*watchful waiting*” approach with a limited reduction in immunosuppressive therapy, if any, and, therefore, a lower risk of allograft rejection.

We report the association between IL28B rs12979860 polymorphism and BKVAN in KT recipients, which supports our hypothesis concerning the role of the IL28B polymorphism as a potential predictor marker for BKVAN progression. Validation studies are needed and if confirmed, IL28B polymorphism could be used to differentiate between patients with self-limited BKV− reactivation and patients with high risk of progression towards BKVAN, and to modulate accordingly the clinical and immunosuppression management of these patients.

## Methods

### Patients

The study conducted at the San Raffaele University Hospital is a single-center retrospective case-control genetic association study whereby renal allograft recipients presenting with acute/chronic graft dysfunction and normal healthy allograft recipients were evaluated. Overall, 30 kidney, 13 kidney-pancreas and 7 double kidney allograft recipients (45 Cadaveric and 5 living related/unrelated HLA matched), who underwent clinical post-transplantation follow-up in our institute, were involved in our study. Written informed consents for the use of clinical data or biological material, for biopsies and for genetic research, were obtained.

### BKV− DNA quantification method and threshold

In our center, all transplanted patients are screened for urine and serum BKV viral load monthly during the first 3 months after transplantation, then every 3 months until the end of the first year, and thereafter annually. BKV− DNA viral load monitoring was carried out with real-time quantitative polymerase chain reaction (qPCR) using the QIAsymphony artus BK Virus QS-RGQ kit (QIAGEN GmbH, Hilden, Germany) targeting part of VP2 and VP3 segments of BKV, according to the manufacturer’s instructions. Although some studies have suggested plasma BKV− DNA level of 10,000 copies/mL as a threshold value to determine BKV infection as significant and insignificant^5, 14^, at our center no cut-off values were set, and a transplant renal biopsy was performed on detection of even lower plasma BKV− DNA viral loads, as later suggested by Hassan *et al*.^34^.

### Allograft biopsy

Patients with serum active BKV− replication and evidence of worsening graft function (persistent reduction of GFR more than 20 ml/min/1.73 mq, or first appearance or worsening of proteinuria) undergo percutaneous echo-guided needle renal allograft biopsy for further histological evaluation. BKVAN diagnosis is based on renal histological findings, according to the Banff Working Classification^40^. All methods were carried out in accordance with our institution guidelines and regulations. All experiments were performed according to protocols approved by the San Raffaele Hospital. Written informed consensus for biopsy was obtained from all patients, while all other tests were part of the “standard of care” in our Institution (IOS -Istruzioni Operative Specifiche- MTRAP 002 RENE E PANCREAS POSTTRAPIANTO – Rev 3).

### IL28B Genotyping

Whole-blood samples were collected in a 7-mL-EDTA BD Vacutainer tube. Genomic DNA was isolated using the QIAamp DNA blood Mini Kit (QIAGEN GmbH, Hilden, Germany), according to the manufacturer’s instructions. The rs12979860 SNP was analyzed using modified invitrogen platinum Taq DNA Polymerase “in-house” PCR protocol. Briefly, 4 µl of DNA was added for a total reaction volume of 50 µL mixture in each sample for PCR amplification. For rs12979860 polymorphism, sequence was obtained from the NCBI Entrez [Reference SNP (refSNP) Cluster Report: rs12979860]. Primers were designed to amplify a total of 278 bp, where polymorphism was assigned in position 130. Primer sequences were as follows: forward 5′-CGG CGC TTA TCG CAT ACG GCT AG-3′; and reverse 5′-ACC CCC GCA GTC CCC GTC-3′. Thermal cycling comprised an initial incubation start for 30 seconds at 94 °C, followed by 35 cycles of denaturation at 94 °C for 30 seconds, annealing at 60 °C for 15 seconds, and extension at 72 °C FOR 60 seconds. The PCR products were visualized on a UV trans-illuminator, followed by gel electrophoresis in 2% agarose gel. The PCR products were purified using the Applied Biosystems by life BIG DYE X terminator PCR purification kit. Single-band PCR product was sequenced using BigDye Terminator DNA-sequencing techniques (Applied Biosystems, Foster city, CA), using both the forward and reverse primers of the amplification PCR reaction. Sequencing data were analyzed using ABI 3130 XL. For rs12979860 SNP, C/C represent the most frequently observed genotypes in Caucasians and Asians (“major” allele), whereas C/T and T/T are termed “minor” allele genotypes.

### Statistical analysis

Data are reported as M + SD. Data were analyzed either with the chi-square or the median test, depending on the level of measurement, using SPSS Inc., Chicago, IL, USA, software. SNP genotyping data were confirmed using SNPStats, a web tool for the analysis of association studies. For all statistical tests, a p-value < 0.05 was considered statistically significant, and all P-values are two-tailed. The Hardy–Weinberg equilibrium was assessed by the Chi-squared statistics [reference - HapMap group in Caucasian population; C/C 0.48, C/T 0.41, T/T 0.11 frequencies in LTG_NCI_NIH-HapMap-CEU (https://www.ncbi.nlm.nih.gov/SNP/snp_ref.cgi?rs=s12979860)].
